# Individual Objective and Subjective Fixation Disparity in Near Vision

**DOI:** 10.1371/journal.pone.0170190

**Published:** 2017-01-30

**Authors:** Wolfgang Jaschinski

**Affiliations:** Leibniz Research Centre of Working Environment and Human Factors, Dortmund, Germany; State University of New York Downstate Medical Center, UNITED STATES

## Abstract

Binocular vision refers to the integration of images in the two eyes for improved visual performance and depth perception. One aspect of binocular vision is the fixation disparity, which is a suboptimal condition in individuals with respect to binocular eye movement control and subsequent neural processing. The objective fixation disparity refers to the vergence angle between the visual axes, which is measured with eye trackers. Subjective fixation disparity is tested with two monocular nonius lines which indicate the physical nonius separation required for perceived alignment. Subjective and objective fixation disparity represent the different physiological mechanisms of motor and sensory fusion, but the precise relation between these two is still unclear. This study measures both types of fixation disparity at viewing distances of 40, 30, and 24 cm while observers fixated a central stationary fusion target. 20 young adult subjects with normal binocular vision were tested repeatedly to investigate individual differences. For heterophoria and subjective fixation disparity, this study replicated that the binocular system does not properly adjust to near targets: outward (exo) deviations typically increase as the viewing distance is shortened. This exo proximity effect—however—was not found for objective fixation disparity, which–on the average–was zero. But individuals can have reliable outward (exo) or inward (eso) vergence errors. Cases with eso objective fixation disparity tend to have less exo states of subjective fixation disparity and heterophoria. In summary, the two types of fixation disparity seem to respond in a different way when the viewing distance is shortened. Motor and sensory fusion–as reflected by objective and subjective fixation disparity–exhibit complex interactions that may differ between individuals (eso versus exo) and vary with viewing distance (far versus near vision).

## Introduction

Seeing with two eyes is an important aspect of human vision. Binocular vision refers to the integration of the images in the two eyes for improved visual performance and depth perception. A comprehensive review of binocularity is provided in the seminal books of Howard [1], Howard & Rogers [2], Howard [3]. One aspect of binocular vision is the fixation disparity, which refers–broadly speaking–to a suboptimal condition in individuals with respect to binocular eye movement control and subsequent neural processing [1], p. 488–492. There are two types of fixation disparity—subjective and objective—that differ in the measurement paradigm, the physiological meaning, and the area of applications. In a recent paper, Schroth et al. [4] have given an extended research overview of the two types of fixation disparity which is summarized here regarding the present study on horizontal fixation disparity in near vision.

**Fig 1:**
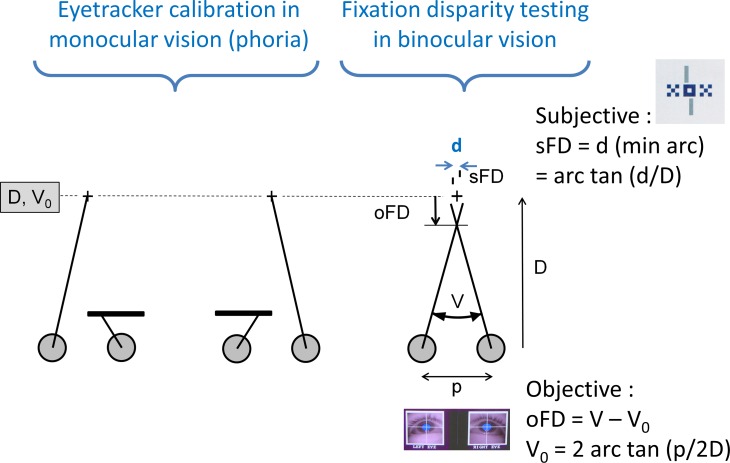
Definition of objective and subjective fixation disparity. Eye position during monocular calibration of each single eye when the fellow eye is occluded and during binocular testing with stimulus shown in the inset, i. e., a central binocular target (XOX) and two vertical dichoptic nonius lines. The geometrically expected vergence angle V_0_ = 2 arc tan (p/2D) depends on the viewing distance disparity and the inter-pupillary distance p so that objective fixation disparity is the resulting vergence error oFD = V − V_0_. Subjective fixation disparity sFD = arc tan (d/D) is given by the angular amount of the nonius offset (Figure adopted from Schroth et al. [4]). In this case, the visual axes cross in front of the target which is referred to as eso fixation disparity with a positive sign. In other cases, the visual axes may intersect behind the target which means an exo fixation disparity with a negative sign. Note that the visual axes do not intersect with the nonius lines, since typically sFD is much smaller than oFD.

The objective fixation disparity refers to the oculomotor position of the eyes, i. e., the vergence angle between the visual axes, which is measured with eye trackers using a monocular calibration procedure: when targets are fixated by the left or right eye alone, they are assumed to be projected onto the centre of the foveola and the corresponding left and right eye positions define a theoretical vergence angle. This is assumed to represent the optimal vergence state, i. e., zero objective fixation disparity (Fig 1). Any deviating vergence state (vergence error) represents an objective fixation disparity, i. e., the images in the two eyes of an object in space are not projected onto corresponding retinal points. Still, the object is not seen double, but single. Neural mechanisms with different stages provide sensory fusion, i. e., the two retinal images are attributed with the same visual direction with respect to the cyclopean eye [1, 2, 5]. If—additionally to the binocular fusion stimulus—physically aligned monocular test stimuli are presented to the right and the left eye (Fig 1), these lines may be perceived with an offset and, thus, appear in different visual directions. Traditionally, this psychophysically measured nonius offset was referred to as subjective fixation disparity with the (false) assumption that it may indicate the vergence position of the visual axes. This application of nonius lines originates from measuring the horopter, i. e., the location of corresponding points in the two retinae. Howard and Rogers [2] p. 173 reviewed this procedure with the conclusion that dichoptic nonius lines—theoretically—are the purest and most reliable measure of binocular correspondence, however several factors can influence the results.

For fixation disparity, the following conditions are important. A clinical nonius application is the test of heterophoria (i. e., the non-fusion vergence resting state), where a point and a line of light serve as dichoptic nonius targets (as in the Maddox procedure); this psychophysical method gives the same vergence angle as eye tracker recordings [6, 7]. However, in conditions of fusion, the psychophysical result of dichoptic nonius lines (subjective fixation disparity) does not agree with the result of eye tracker recordings (objective fixation disparity). This nonius artifact was revealed in different experimental paradigms: (a) dichoptic nonius lines gave different results than the psychophysical phenomenon of the bandwidth of border enhancement that varies with retinal eccentricity, but does not depend on retinal correspondence [8–10]; this was measured as a function of static forced vergence, i. e., changes in vergence that were induced by absolute disparity at a fixed viewing distance, thus at a fixed accommodative stimulus; (b) dichoptic nonius lines gave also different results as eye tracker recordings in static forced vergence [11–14]; (c) dynamic forced vergence changes were not indicated by a monocular line [15]; (d) the disparity offset of two stereoscopic depth planes was not indicated by two pairs of nonius lines [16]; (e) the nonius artifact increased the closer the nonius test was located to the binocular fusion target [16–20]; (f) when forced vergence was not applied, individual measures can still be about 10 times larger for objective fixation disparity than for subjective fixation disparity [4, 21]. For the interpretation of these effects, partly different terminology was used: changed relation between retinal location and perceived visual direction, shifts in retinal correspondence, remapping of visual directions, capture of visual direction. But, the common conclusion of these different approaches is that the visual direction of monocular nonius lines are not transferred unaltered to the cyclopean eye, which would be required for a valid nonius vergence indicator [16]. The subjective fixation disparity seems to be affected by two processes: the oculomotor adjustment of the visual axes (objective fixation disparity) and the remapping of visual directions by sensory fusion [9, 22]. This may explain why subjective and objective fixation disparity were correlated and each measure was correlated with heterophoria [4, 21].

These previous researches showed that subjective fixation disparity does not exactly agree with objective fixation disparity, as reviewed by Howard [1], pp. 488–495. Therefore, different definitions have to be applied. Objective fixation disparity is the vergence error, i. e. oFD = V–V_0_ with V_0_ = 2 arc tan (p/2D), which refers to the position of the visual axes (see Fig 1 with the inter-pupillary distance p and the viewing distance D). If one would assume that dichoptic nonius lines with an offset d would indicate the vergence error, the subjective fixation disparity would be defined as sFD = 2 *(arctan((p + d)/2D)—arctan(p/2D)). However, since measures of oFD and sFD differ and, thus, nonius lines do not indicate the vergence angle, subjective fixation disparity is defined here simply as the angular amount of the nonius offset, i. e. sFD = arctan (d/D). This definition does not refer to the position of the visual axes, rather reflects the angular nonius separation on the cortical level, similar as a binocular disparity of stereoscopic images in the two eyes. The distinction between these two definitions was made here in order to emphasise the different nature of the two types of fixation disparity. But note that a binocular disparity corresponds to an equivalent change in vergence so that the numerical results of these two definitions of sFD differ only by less than 2% in the present conditions and therefore can be compared quantitatively.

Until now, the discrepancy between objective and subjective fixation disparity could only be explained by the rather general assumption of changes in retinal correspondence. Still today, “the intriguing question is what the nonius displacements represent or are a measure of”, as already stated by Kertesz and Lee [11]. Interestingly, these basic researches (that started around 1985) were preceded by clinically-oriented researches [23–26] which provided evidence for the practical relevance of subjective fixation disparity since it was related to asthenopic complaints. Thus, nonius test results belong to the repertoire of clinical management in cases of binocular disorder as described in the textbooks of Evans [27] and Scheimann and Wick [28]. Wick [22] commented on the clinical use of dichoptic nonius lines that “given the clinical success … that management based on fixation disparity measures enjoys, it seems imprudent to discount the clinical research concerning the use of fixation disparity measures…”. Thus, there is a gap between the established clinical nonius application and the uncertainty of the physiological meaning of subjective fixation disparity revealed by research studies.

Previous research was mostly confined to laboratory test conditions as forced vergence, multiple depth planes or vergence dynamics, while conditions of normal everyday visual tasks were hardly investigated. But these normal viewing conditions may be important to explain asthenopic symptoms that occur in some subjects, particularly in near vision. The most common visual task is reading of text and, interestingly, the reading research community has conducted a series of studies on fixation disparity during reading [29–39]. However, the short reading fixations do not allow simultaneous tests of subjective fixation disparity which is important for the optometric aspects of fixation disparity. Therefore, the present study of objective and subjective fixation disparity uses the condition of stationary fixation when nonius lines can be superimposed on a central fusion stimulus in near vision.

Near vision is an important test condition in clinical optometry. Subjects with stronger asthenopic complaints tend to have a larger exo subjective fixation disparity in near vision (40 cm), but not in far vision (5 m) [24, 40, 41]. Research studies varied the viewing distance in smaller steps and to describe a slope of subjective fixation disparity as function of viewing distance, referred to as proximity curve [42, 43]. This slope differs between observers: those with steeper slopes (i. e., larger exo subjective fixation disparity at near) tended to indicate higher levels of asthenopic complaints and preferred longer viewing distance, where their subjective fixation disparity was smaller [44]. Thus, an exo state of subjective fixation disparity appears to be a less favorable binocular condition. Concerning objective fixation disparity, there appears to be only one study that varied the vergence state as in natural vision, i. e., by presenting targets in free space at different viewing distance: Erkelens et al. [45] reduced the viewing distance from 91 to 10 cm and found in their three observers that objective fixation disparity was generally exo and became more exo following a parabolic function as the viewing distance was shortened; subjective fixation disparity was not measured.

Previous research did not investigate the two types of fixation disparity in near vision. Therefore, this was made in the present study during stationary fixation of a central fusion target at viewing distances of 40, 30, and 24 cm from the eyes, in order to see the expected changes in fixation disparity. Repeated sessions were applied to test individual differences in the effects within a sample of 20 young adult observers with normal binocular vision.

## Methods

Measurements of objective fixation disparity in near vision at viewing distances of 40, 30, and 24 cm pose considerable methodological requirements on display technology, eye movement recordings and data analyses. Important features of the present approach are (1) the use of a purpose-made high resolution OLED-display to present sharp images even at the 24 cm viewing distance and to operate shutter glasses for diochoptic nonius lines, (2) elaborate procedures for precise recording binocular eye movements with the video eye tracker EyeLink II that detects the position of the pupil centre, (3) correction for the artifact that the pupil center shifts nasally if the pupil shrinks when the viewing distance is shortened from 40 to 24 cm and when vision changes from monocular calibration to binocular recording. These methodological issues and particularly the correction of the pupil artifact were described by the author [46] in a parallel paper on the same dataset; the present methodological description is confined to the general structure and design of this experiment.

### Participants

The 20 young adult subjects aged 19–31 years (25 ± 3.5, mean ± SD) and had normal vision without eye glasses. The far visual acuity—in decimal units—was 1.0 or better: 1.5 ± 0.2 and 1.6 ± 0.3, in the left and right eye, respectively. The accommodative near point was 15 cm or better: 12.9 ± 1.8 cm and 12.7 ± 1.6, in the left and right eye, respectively. Thus, the subjects had clear vision at the near displays. The participants had a threshold of stereo vision of 2 min arc or better (median 1 min arc) tested with the TNO test using the circles with missing sectors. This research was approved by the ethic committee of the Leibniz Research Centre of Working Environment and Human Factors (IfADo); the procedures were in accordance with ethic practice and participants signed a written consent.

### Study design

Two experimental sessions were made on different days. Each session comprised twelve runs in random order: four repeated runs at each of the three viewing distances of 40, 30, and 24 cm, which were chosen to have equidistant vergence angles of 9, 12, and 15 deg; equidistant x-values are advantageous for regression analyses. These angles were divided by the vergence angle at a 1 m viewing distance, i. e., 3.6 deg at a 6.3 cm inter-pupil-distance. This leads to the vergence conditions of 2.5, 3.3, and 4.2 meter angle; the latter unit of vergence is convenient since it represents the inverse of the viewing distance in meters (1/meter). It is known that fixation disparity is linearly related to the vergence angle [43]. Viewing distances in the range of 40–24 cm were chosen so that the participants were able to hold their hand at the display; this was made in order to investigate whether vergence accuracy may be improved if observers hold their hands at the visual display. Effects of hand-proximity are known for cognitive functions, depending on test conditions [47, 48]. Therefore, in half of the runs in the present experiment, participants operated response buttons at the display and in the other half they operated a computer mouse hold on their lap. This manipulation, however, did not have an effect on fixation disparity (t = 0.2, p = 0.42, df = 19). Therefore, this factor is ignored in the present paper.

### Recording of the dependent measures

**Fig 2:**
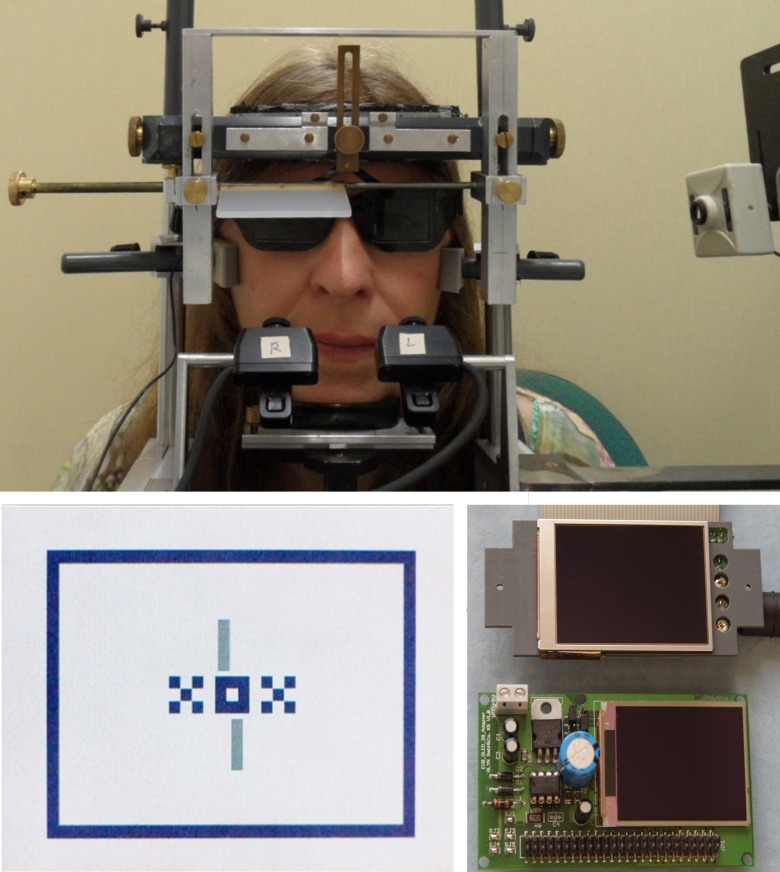
Illustration of experimental setup and OLED display. **Upper** photograph: Observer in the adjustable headrest with rests for the chin and the forehead; the cheekbones were fixed to prevent horizontal head movements; a flexible band around the head held the observer in the headrest. Shutter glasses provided dichoptic viewing of the nonius lines. The EyeLink II cameras had an unobstructed view of the eyes below the shutter glasses. The knob (at the left) allowed moving an occluder in front of each eye for the monocular calibration. The side-view camera (at the right) gave a video image that allowed placing the eye at the correct position with respect to viewing distance and height. Left lower photograph: visual stimulus with a central and peripheral binocular fusion target (XOX and outer frame) and dichoptic nonius lines¸ a single nonius line subtended 43 min arc. Right lower photograph: Two types of raw electronic devices with OLED displays before installation into the experimental setup.

Each run consisted of a 1-minute recording period during which the subject fixated a target (inset in Fig 2) that comprised a central and peripheral binocular fusion target (XOX, surrounded by a frame) and two nonius lines. The nonius lines were presented dichoptically, i. e., the upper line to the right eye and the lower to the left eye. The subjects operated a computer mouse in order to shift the nonius lines horizontally relative to each other until they appeared in alignment. A mouse click indicated the perceived aligment and initiated a randomly varied physical nonius offset for the subsequent alignment. The resulting nonius offset was recorded as a single measure of the subjective fixation disparity and—synchronously—the corresponding pupil size and binocular eye position was recorded. During one run of 1 minute, the observers typically made a series of 10 to 20 nonius adjustments. This is also the number of single recordings of objective fixation disparity and pupil size.

### Display

The stimuli were presented on an OLED-Display (CMEL, diagonal size 2.4 inch, resolution 240 x 320 pixel, RGB, pixel pitch 0.051 x 0.153 mm). This OLED-display system is not a commercial monitor; rather the control software and hardware, including the graphics card were developed in our institute for the present research (Fig 2). The OLED display and shutter glasses (Elsa Revelator) were controlled by a purpose-made graphic board to present different stimuli to the two eyes which is necessary for dichoptic nonius lines. The frame rate of the display was 140 Hz so that the shutter glasses could be operated at 70 Hz for each eye, which prevented visible flicker at the given screen size and luminance. Shutter glasses have the limitation of crosstalk, i.e. the right eye perceives a small proportion of the image for the left eye (and vice versa) so that each eye may perceive a faint nonius line of the fellow eye. This was avoided by using a white background on which a black line due to crosstalk remains below the luminance threshold (on a black background, even a faint white line from the fellow eye would be visible). Technically, the extent of crosstalk depends on the type of shutter glasses and monitor and their mode of operation. Crosstalk can be quantified by—rather difficult—measurements of very low luminance increments. Still, such physical measurements would not be sufficient, since the crucial criterion is that effects of crosstalk are not perceived by the observer. In the present setup, effects of crosstalk were not visible.

The OLED display was fixed on a mechanical slide that allowed for precise adjustment of the viewing distance from the centre of the eye. The size of the stimulus was adjusted to have the same angular dimensions at the viewing distances of 24, 30, and 40 cm: a single nonius line subtended 43 min arc. The background luminance was 6 cd/m^2^, measured through the shutter glasses. The OLED-Display was surrounded by a white board (20 cm square) of similar luminance, provided by light panels. The ambient room illumination was constantly dim at about 10 lx in the laboratory without windows. The experimental setup and the OLED display are illustrated in Fig 2.

### Eye movement recordings

The video-based EyeLink II (SR Research Ltd, Osgoode ON, Canada) was used with the dark pupil detection mechanism that tracks the centre of the pupil. Recorded data were analyzed based on the raw data, sampled at a rate of 2 ms (500 Hz). The filters of the EyeLink software were switched off.

The conventional EyeLink II procedures were modified in order to improve performance for measuring fixation disparity [21]; the accuracy of the present recording and measurement approach are described in Jaschinski [46]. A chin and forehead rest, a band around the head and narrow temporal rests were applied to minimize artefacts due to possible lateral and oblique head movements; a bite bar was not used. The headrest could be adjusted very flexible in order to place the eye at a defined position for all subjects. This correct eye position was controlled by a video camera beside the head. Such precise adjustments are important at the short viewing distances in this experiment. The two EyeLink II cameras were fixed to the headrest.

Instead of the original EyeLink II calibration mode, we used the raw data and applied the following monocular calibrations before and after the 1-minute recording period that were averaged. The use of shutter glasses is not sufficient for complete monocular vision during the calibration since the mechanical frame of the OLED display may be effective as peripheral fusion target. Therefore, the right eye was covered with an opal occluder for calibrating the left eye and, subsequently, the left eye was covered for calibrating the right eye. The opal occluder was chosen to make all stimuli invisible, but to lower the luminance by only 30% so that the pupil dilated only slightly due to the occlusion. For calibration, subjects were requested to carefully fixate one of three calibration targets (crosses of 10 min arc) that appeared sequentially in the screen centre (zero position) and deviating horizontal positions at 120 min arc. Each of the three calibration targets was presented three times in random order to be able to average across variability in fixation.

### Measurement of accommodation

Fixation targets were presented at viewing distances of 120, 60, and 30 cm; the left eye accommodation was measured with a free-view autorefractor SRW 5000 (Shin Nippon). The AC/A-ratio is the amount of convergence change that is induced by a change in accommodative response, i. e., accommodative convergence (AC) per accommodation. For measuring the accommodative convergence, a small red point light source was attached near the fixation target for the left eye and this appeared as a vertical streak of light for the right eye that was covered by a Maddox rod (strong vertical cylindrical lens). A rotary prism in front of the right eye was used for measuring the fusion-free vergence angle (subjective heterophoria). A regression of the heterophoria as a function of accommodative response was calculated across the three viewing distances of 120, 60, and 30 cm; the resulting slope is the AC/A-ratio. These measurements were made in a vision screening session some weeks before the two main sessions.

### Data analyses and statistical analyses

Video eye tracker detect the centre of the pupil, but these recordings of eye position can be affected by an artefact since the centre of the pupil can shift horizontally and vertically when the pupil size varies at constant eye position. This pupil artefact is relevant in the present study since the pupil shrinks with increasing near vision when the viewing distance is shortened from 40 to 24 cm and, further, the pupil is smaller during the 1-minute recording with binocular vision than during the calibration with monocular vision. This artefact was corrected for the present study with a procedure described in a separate paper [46]. In short, a regression was calculated to predict the objective fixation disparity from the difference in pupil size between calibration and test phase. This provides—for each viewing distance—an estimation of objective fixation disparity that corresponds to the same pupil size during the test and during the calibration. In this way, the data in the present paper are corrected for the pupil artefact. The average of the series of nonius adjustments within the 1-minute recording period gave the subjective fixation disparity of a run.

The open-source software R (The R Foundation) was applied [49]. Fixed effects of viewing distance and random effects of subjects were tested with linear mixed-effects models (LMER) [21, 50, 51, 52]. Relations between different vergence and accommodative measures were tested with robust regressions (LMROB) to reduce the influence of outliers; one-tailed *p*-values were used since all hypotheses were one-directional. The complete dataset of the individual subjects is displayed in S1 Fig and the statistical procedures are described in S1 File.

## Results

The results comprise three steps. First, the averages in the group are presented as a function of viewing distance. Second, mean effects and individual differences are tested by linear mixed-effects models. Third, the data of each participant are described by the individual mean fixation disparity and the individual change in fixation disparity with viewing distance; the reliability of these data between repeated sessions is reported. Fourth, inter-individual regression analyses are made of the observers’ objective versus subjective fixation disparity. Finally, it was tested whether the inter-individual variance in the two types of fixation disparity can be explained by the observers’ heterophoria or accommodative performance.

### Mean results of fixation disparity versus viewing distance

**Fig 3:**
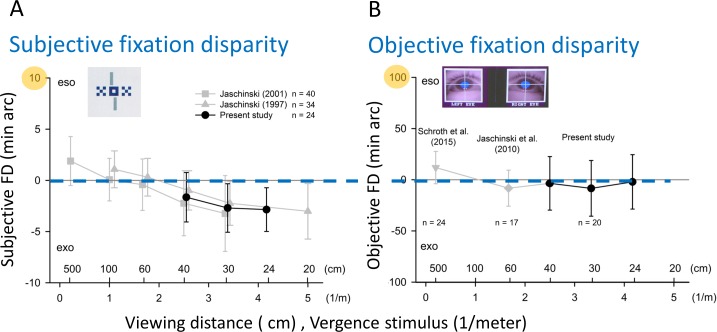
Mean subjective and objective fixation disparity as a function of viewing distance. Viewing distance is given in the unit “1/meter”, which corresponds to the unit “meter angle”, i. e., a vergence angle between the visual axes, normalized to the vergence angle of 3.6 deg at the viewing distance of 1 meter. Positive and negative values mean an over-convergence (eso) or under-convergence (exo), respectively. Note that the scale is ten times larger for objective than for subjective fixation disparity and that the maximal values of the scales were chosen to agree with the larger ranges of the scatter of individual values in Figs 4–8. The black symbols at the viewing distances of 40, 30 and 24 cm refer to the data in the present study; for comparison, the grey symbols refer to results of previous studies (see Discussion).

Fig 3 shows the group means of objective and subjective fixation disparity as a function of viewing distance; the latter is described in the unit “1/meter” correspondings to the unit “meter angle”, which is a vergence angle, normalized to the vergence angle of 3.6 deg at the viewing distance of 1 meter. This unit is favorable since 1/meter is a conventional unit in optometry and since it provides linear changes in fixation disparity with the vergence angle. On a first glance, the two types of fixation disparity may seem to have similar mean values, but with a much larger standard deviation for objective fixation disparity. However, the important marked difference is revealed by the repeated-measures statistics described in the next section: the mean subjective fixation disparity is clearly negative (exo) and changes to a more exo condition as the viewing distance is shortened from 40 to 24 cm (Fig 3A), while the mean objective fixation disparity is close to zero and does not change with viewing distance (Fig 3B). Note that the scale is ten times larger for objective than for subjective fixation disparity; thus, the individual amount is much larger for objective than for subjective fixation disparity.

### Quantifying individual differences with mixed-effects models

A systematic statistic approach to quantify individual differences are mixed-effects models that include random effects to account for the data variability that can be attributed to differences between subjects; fixed effects of experimental conditions, as viewing distance, are also included. In mixed-effects modeling, several models of increasing complexity are defined and the additional advantage of the increased model complexity is tested stepwise. This statistical procedure is described in the Supporting Information S1 File and results are summarized in Table 1. The first analysis refers to the objectively measured heterophoria, i. e., the vergence angle in the non-fusional state during the calibration. This was made since heterophoria is an established clinical measure of vergence, so it may be informative to see how the common knowledge about heterophoria shows up in this mixed-effects modeling. This provides a comparison with the following models for subjective and objective fixation disparity.

**Table 1:** Series of mixed-effects models (A, B, C, D) with increasing complexity for the dependent variables heterophoria, subjective and objective fixation disparity. The goodness of fit of a model is given by the information criteria AIC and BIC; model improvements are tested by ANOVAs. Intercept (at the centered 30 cm viewing distance) and fixed effects are described by the mean ± standard error. Random effects are described by the Repeatability Index R_i_ = SD_inter_ /SD_intra_, the ratio of inter- to intra-individual standard deviation. 120 observations were included (20 subjects, 2 sessions, 3 viewing distances); the 4 repetitions per condition were averaged in beforehand. The best model is highlighted: Model D for heterophoria, Model C for subjective and Model B for objective fixation disparity. If cells in the table are empty, these effects were not included in the corresponding model.

**Heterophoria**											
**Model**	**Intercept**			**Fixed effect of viewing distance**			**Random subject effects**		**AIC**	**BIC**	**Model testing**
							**Intercept**	**Viewing distance**			
	**Mean ± SE**	**t—value**	**p -value**	**Mean ± SE**	**t—value**	**p -value**	**SD**_**inter**_ **/ SD**_**intra**_	**SD**_**inter**_ **/ SD**_**intra**_			**p–value**
	**(deg)**			**(deg/ma)**							
A	-4.03±0.22	18.14	<0.001						560	568	
B	-4.02±0.48	8.31	<0.001				2.08/0.22 = 9.45		427	441	<0.001
C	-4.02±0.48	8.36	<0.001	-1.12±0.07	16.08	<0.001	2.14/0.69 = 3.01		313	330	<0.001
D	-4.02±0.48	8.34	<0.001	-1.12±0.11	9.58	<0.001	2.11/0.77 = 2.74	0.48/0.77 = 0.62	284	309	<0.001
**Subjective fixation disparity**											
**Model**	**Intercept**			**Fixed effect of viewing distance**			**Random subject effects**		**AIC**	**BIC**	**Model testing**
							**Intercept**	**Viewing distance**			
	**Mean ± SE**	**t—value**	**p -value**	**Mean ± SE**	**t—value**	**p -value**	**SD**_**inter**_ **/ SD**_**intra**_	**SD**_**inter**_ **/ SD**_**intra**_			**p–value**
	**(min arc)**			**(min arc/ma)**							
A	-2.39±0.22	10.8	<0.001						559	568	
B	-2.47±0.49	5.08	<0.001				2.32/1.01 = 2.32		431	445	<0.001
C	-2.47±0.49-	5.09	<0.001	- 0.73 ± 0.10	7.09	<0.001	2.35/1.12 = 2.10		394	410	<0.001
D	-2.43±0.49-	5.00	<0.001	- 0.73 ± 0.13	5.38	<0.001	1.60/0.66 = 2.42	0.45/0.66 = 0.68	393	418	n. s
**Objective fixation disparity**											
**Model**	**Intercept**			**Fixed effect of viewing distance**			**Random subject effects**		**AIC**	**BIC**	**Model testing**
							**Intercept**	**Viewing distance**			
	**Mean ± SE**	**t—value**	**p -value**	**Mean ± SE**	**t—value**	**p -value**	**SD**_**inter**_ **/ SD**_**intra**_	**SD**_**inter**_ **/ SD**_**intra**_			**p–value**
	**(min arc)**			**(min arc/ma)**							
A	-4.68±2.63	1.77	0.08						1153	1162	
B	-4.33±5.25	0.82	0.41				22.34/16.95 = 1.32		1060	1074	<0.001
C	-4.33±5.25	0.82	0.41	-0.86±1.83	0.47	0.63	22.34/16.96 = 1.32		1061	1078	n. s.
D	-4.32±5.25	0.82	0.41	-0.86±2.50	0.34	0.73	16.02/17.98 = 0.89	8.78/17.98 = 0.49	1059	1084	n. s.

In Table 1, Model A represents the–unrealistic–null model assuming that heterophoria is the same in all subjects and at all viewing distances; all variance in the dataset is assumed to be due to random error. This model indicates a mean ± SE of heterophoria of – 4.02 ± 0.21 deg. This null model is required as a reference for Model B which is more realistic since it includes the well-known individual differences in heterophoria. Individual differences are large, if the heterophoria differs considerably between subjects, but does not change much within a single observer in repeated tests. As repetitions, the mean results of each of the two sessions were used. Corresponding standard deviations between subjects (SD_inter_) and within subjects (SD_intra_) are provided by a random subject effect in Model B which gives a significantly better fit to the data than Model A, as indicated by significantly lower information criteria AIC and BIC. However, Model B does not provide the final results since further model improvement are possible. In the next step, Model C additionally assumes—as a fixed effect—that the heterophoria may depend on viewing distance (to the same extent in all subjects); physiologically, one should expect a shift to more exo states as the viewing distance is shortened. In fact, Model C provides a significantly better fit than Model B and the included fixed effect of viewing distance is significant with an amount of – 1.14 ± 0.09 deg /(1/meter), i. e., the heterophoria becomes more exo by 1.14 deg if the viewing distance is shortened by 1 (1/meter). In the next step, Model D includes the additional assumption that the effect of viewing distance may depend on subjects; again, this model gives a significantly better fit than Model C. Thus, in the series of these four models with increasing complexity, Model D reaches the significantly lowest information criteria (AIC = 284, BIC = 309) and is therefore appropriate for heterophoria. According to Model D, the ratio of SD_inter_ (between subjects) relative to SD_intra_ (within subjects, between sessions), i. e., the so-called repeatability index is R_i_ = SD_inter_ / SD_intra_ = 2.11 / 0.77 = 2.74. Thus, heterophoria varies between subjects 2.7 times as much as within subjects. The fixed effect of viewing distance remains virtually the same as in Model C. Note that Model B and Model C showed higher repeatability indices, but were less appropriate for the dataset due to larger AIC and BIC values.

The same reasoning is applied to fixation disparity; however, the resulting structure of the models is less complex. Subjective fixation disparity gave a similar result as heterophoria, except that Model D was not better than Model C (Table 1). According to the most appropriate Model C, the grand mean subjective fixation disparity was – 2.46 ± 0.44 min arc, which represents the expected exo objective fixation disparity in near vision. The significant fixed effect means that subjective fixation disparity depends on viewing distance with a significant slope of – 0.73 ± 0.10 min arc /(1/meter). This slope is reflected in Fig 3A in the range of 40 to 24 cm. The random subject effect for subjective fixation disparity shows a repeatability index of R_i_ = SD_inter_ / SD_intra_ = 2.35 / 1.12 = 2.10.

For objective fixation disparity, a different pattern of results appeared (Table 1). Model D was not better than Model C and—most important—Model C was not better than Model B, i. e., the fixed effect of viewing distance was insignificant; the negligible mean slope of 0.20 ± 1.96 min arc/(1/meter) corresponds to the flat curve in Fig 3B. Since Model B is significantly better than Model A, the only significant effect in objective fixation disparity was the random subject effect with a repeatability index R_i_ = SD_inter_ / SD_intra_ = 22.34 / 16.95 = 1.32. Further, it is important to note that the intercept was insignificant: the average objective fixation disparity (- 4.33 ± 5.25 min arc) did not differ significantly from zero.

The mixed-effects models provide two main conclusions. (1) Regarding the group average, the subjective fixation disparity is exo and becomes more exo as the viewing distance is shortened, while the mean objective fixation disparity is close to zero and does not change with viewing distance. (2) Individual differences were identified for the amount of subjective and objective fixation disparity, but not for the slope as a function of viewing distance. The apparent differences between objective and subjective fixation disparity are further explored based on correlations of the individual amount of fixation disparity.

### Reliability between sessions

**Fig 4:**
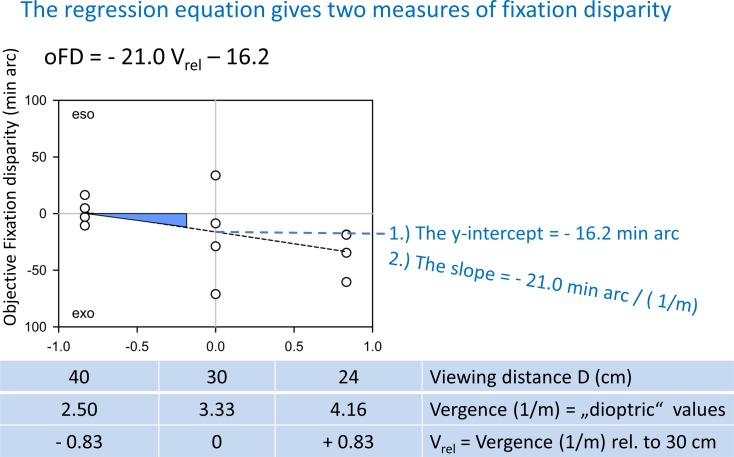
Objective fixation disparity as a function of viewing distance for one session of an individual observer. Viewing distance is plotted in the unit “1/meter” (see Fig 3). For each viewing distance, the four data points of the four runs per session were plotted and used to calculated a robust regression line that reduces the influence of outliers. The resulting y-intercept and slope are indicated.

Fig 4 shows how the data of each individual were aggregated in order to test the reliability between sessions and to describe the results in the two sessions separately. One session comprised 4 runs for each of the three viewing distances. Each run provided a mean subjective fixation disparity based on the series of nonius adjustments and corresponding values of objective fixation disparity. The 12 data of one session were used in robust regression analyses that reduced the effect of possible outliers; the x-values were the vergence angles in the unit “1/meter”, but centered at 30 cm, i. e., 3.33 (1/meter) so that the intercept coefficient of the regression represents the average fixation disparity of all viewing distances of 40, 30, and 24 cm. The slope indicates the change in fixation disparity with viewing distance and has the unit “minute of arc / (1/meter)”. Table 2 provides a comparison of the aggregated data of the two sessions. The mean values of the two sessions (calculated as intercept in the robust regression in Fig 4) showed highly significant test-retest correlations of 0.93, 0.80, and 0.73 (p < 0.001) for heterophoria, subjective and objective fixation disparity, respectively. The corresponding test-retest correlations of the slopes as a function of viewing distance were lower with 0.73, 0.38, 0.50, but still significant (p ≤ 0.05). This suggests that—in terms of the test-retest correlation—the mean fixation disparity is more reliable than the slope in the present dataset. This was already reflected in the mixed-effects model in Table 1, which showed a significant subject effect for the intercept, but not for the effect of viewing distance. Still, the latter slope of objective fixation disparity had a significant test-retest correlation of 0.50, while the mean slope was virtually zero as shown in Fig 3 and Tables 1 and 2. The parallel methodological paper of Jaschinski [46] includes a figure of this test-retest correlation which tentatively suggests that some subjects had a positive slope and others had a negative slope, so that the average slope was close to zero. However, this finding requires further investigations with measurements over a wider range of viewing distances for more reliable slope measures. In the present dataset both this test-retest correlation and the mixed-effects models suggest that the more reliable measure is the fixation disparity intercept, estimated at the 30 cm viewing distance. This measure is therefore used for the subsequent regression analyses between different vergence (and accommodative) measures.

**Table 2:** Comparison of the two sessions with test-retest correlations and Bland-Altman analyses with respect to the mean value at 30 cm and the effect of viewing distance: For heterophoria (a), subjective (b) and objective fixation disparity (c) it is displayed (1) the group mean values ± SD of the two sessions, (2) the robust test-retest correlations (p ≤ 0.05 for r ≥ 0.38) and (3) mean differences ± SD between sessions.

		Mean at 30 cm			Effect of viewing distance		
		Intercept of robust regression			Slope of robust regression		
		Mean ± SD	Test-retest correlation	Difference ± SD between sessions	Mean ± SD	Test-retest correlation	Difference ± SD between sessions
**(a) Heterophoria**	**Session 1**	-4.0±2.2	0.93	0.02±0.78	-1.17±0.51	0.73	0.04±0.39
**(deg)**	**Session 2**	-4.0±2.4			-1.13±0.55		

**(b) Subjective fixation disparity (min arc)**	**Session 1**	-2.3±2.4	0.80	- 0.20±1.20	-0.84±1.11	0.38	0.14±1.04
	**Session 2**	-2.5±2.3			-0.69±0.54		
**(c) Objective fixation disparity (min arc)**	**Session 1**	-3.9±24.5	0.73	- 0.01±20.52	- 0.92±14.8	0.50	2.80±14.43
	**Session 2**	-3.9±29.2			- 1.9±11.5		

### Relation between different individual measures

**Fig 5:**
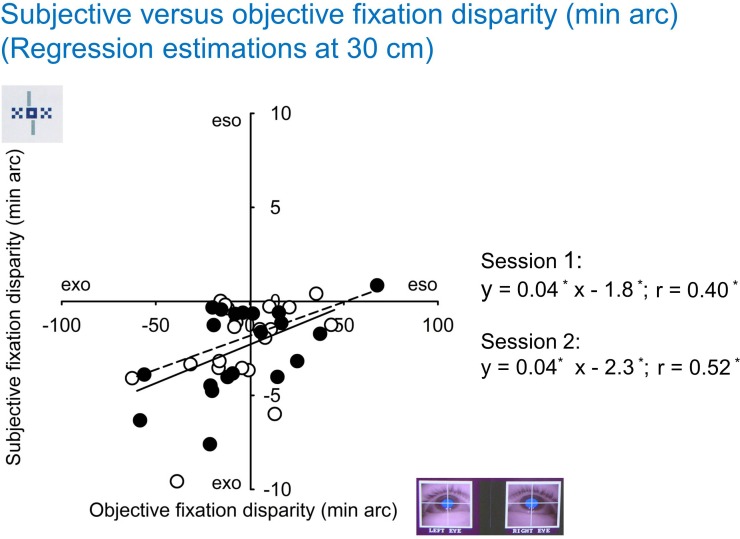
Robust regression between subjective and objective fixation disparity (intercept at 30 cm). The results of Session 1 and Session 2 are shown separately by open and closed symbols and by broken and drawn lines, respectively. In the regression equations, asterisks indicate significant coefficients.

Fig 5 shows the scatter plot of subjective fixation disparity as a function of objective fixation disparity, based on the intercepts of individual robust regression. The two sessions were analyzed separately and gave similar results. The individual subjective fixation disparity was negative (exo) in almost all cases, which is plausible in near vision. Surprisingly, in many cases the objective fixation disparity was positive (eso), while the corresponding subjective fixation disparity was negative (exo). All regression coefficients and correlation coefficients were significant, meaning that the two types of fixation disparity are correlated. However, the proportion of explained variance is limited (up to 25%). The regression lines of both sessions suggest that at oFD = 0 the corresponding sFD is 2 min arc.

**Fig 6:**
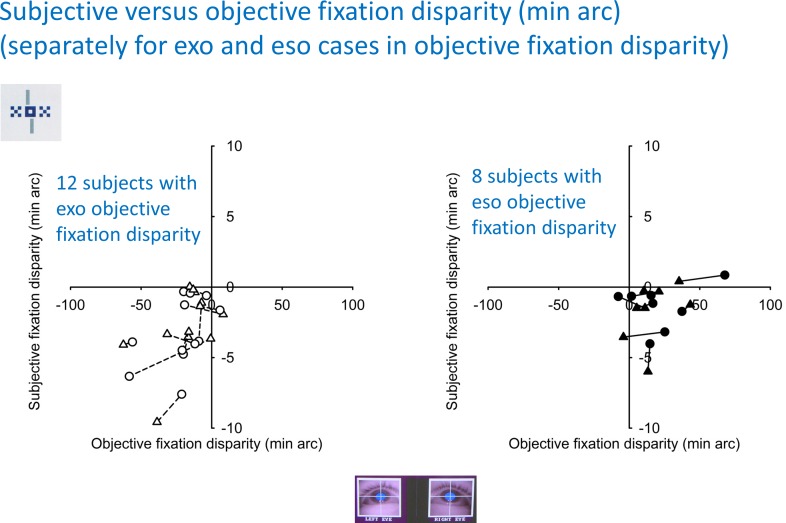
Replotted data of subjective versus objective fixation disparity (see Fig 5). Lines connect data points of Session 1 and Session 2 for each subject to illustrate the similarity of these two results; triangles refer to Session1 and circles to Session 2. Two subgroups were formed based on whether the mean objective fixation disparity was negative (exo, n = 12) or positive (eso, n = 8).

The same data were plotted in a different way in Fig 6. The two data points of a single subject (from Session 1 and Session 2, respectively) were connected by a line to illustrate the variability within a subject. Many subjects gave rather similar data in the two sessions, some had more different data. This plot was made separately for 12 subjects with an exo objective fixation disparity (left graph) and for 8 subjects with an eso objective fixation disparity (right graph). Note that these two subgroups have separate clusters of data points in the lower left quadrant and lower right quadrant. There appears to be a subgroup of 7 subjects in the lower right quadrant with reliably different signs of fixation disparity. The following analyses try to identify physiological conditions of the latter cases.

**Fig 7:**
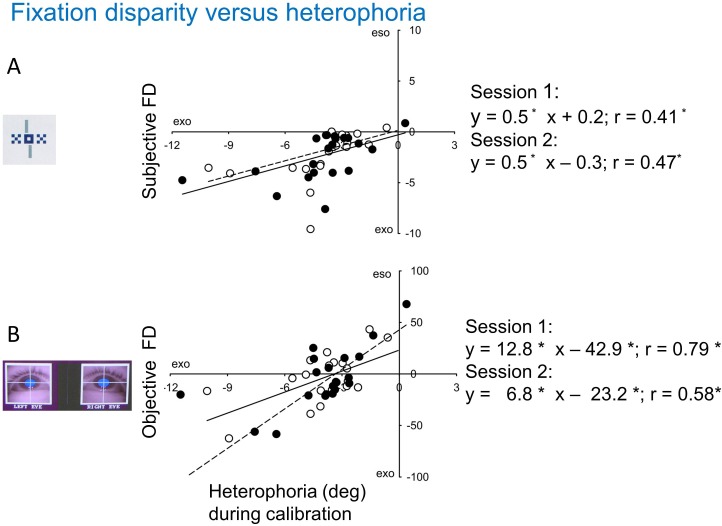
**Robust regressions showing the effect of heterophoria on subjective (A) and objective (B) fixation disparity (intercept at 30 cm).** The results of Session 1 and Session 2 are shown separately by open and closed symbols and by broken and drawn lines, respectively. In the regression equations, asterisks indicate significant coefficients.

Fig 7 shows significant correlations between heterophoria and the two types of fixation. For subjective fixation disparity, the intercept is close to zero meaning that a heterophoria of zero is associated with a subjective fixation disparity of zero (as it can be expected). For objective fixation disparity, however, the intercept is significantly positive meaning that a zero heterophoria is associated with an objective fixation disparity of 33 min arc, on the average. Surprisingly, in the latter regression the slopes differ between the two sessions, however no reason for this discrepancy is apparent. Still, both sessions agree in the finding that the y-intercept and x-intercept deviate from the origin.

**Fig 8:**
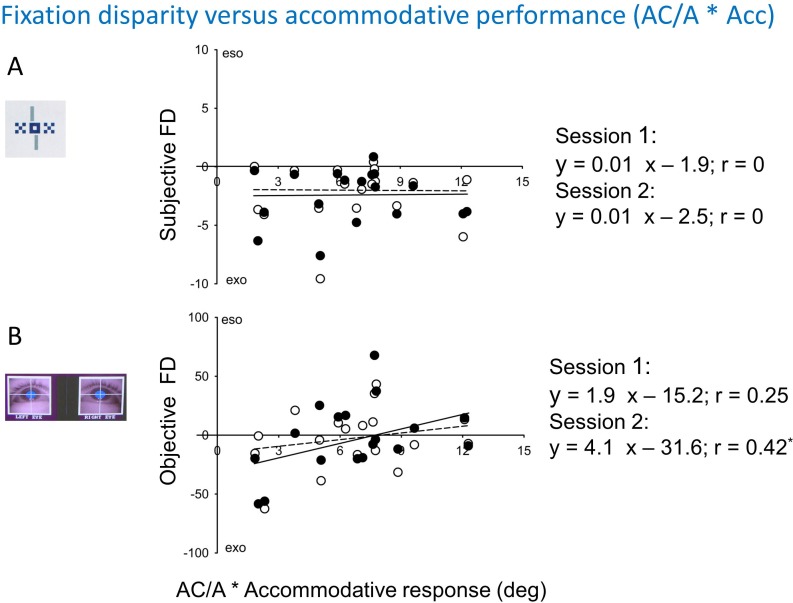
**Robust regression showing the effect of accommodation on subjective (A) and objective (B) fixation disparity (intercept at 30 cm).** The accommodative performance is the product of the AC/A-ratio and the accommodative response at 30 cm. The results of Session 1 and Session 2 are shown separately by open and closed symbols and by broken and drawn lines, respectively. In the regression equations, asterisks indicate significant coefficients.

Fig 8 illustrates the influence of the subject’s accommodative performance on their individual fixation disparity; as accommodative measure, it is used the product of the response AC/A ratio times the accommodative response at a 30 cm viewing distance; this measure represents the vergence angle in deg that is thought to be exerted from accommodation alone. For subjective fixation disparity, both sessions revealed that there is no correlation in these inter-individual correlations; note that intra-individual changes in accommodations will obviously induce a vergence shift via the coupling of accommodation and vergence within a subject. For objective fixation disparity, a weak influence of inter-individual difference in accommodation appeared: the correlation in Session 2 was significant, however both slopes–although being positive–were insignificant. In fact, this correlation was not high, may be because only 18 of all 20 participants were available for this test and the accommodative measurements have not been made simultaneously with the eye movement recordings, but some weeks earlier.

## Discussion

The discussion comprises several steps. First, the results concerning subjective and objective fixation disparity are discussed separately in comparison with previous research. Second, the difference between subjective and objective fixation disparity and the relation between the two are interpreted. Third, based on these results, an outlook to further research is provided.

### Fixation Disparity as a Function of Viewing Distance

The present findings regarding subjective fixation disparity as a function of viewing distance (40–24 cm) agree well with results of several earlier studies that covered a much larger range of viewing distances (5 m–20 cm) [42, 43]. These later two studies and the present study are included in Fig 3A since the experimental conditions were similar, since a central fusion stimuli was used. Thus, Fig 3A illustrates the proximity curve in subjective fixation disparity that shifts from an eso state in far vision to an exo state in near vision. Similar exo proximity effects were found for subjective fixation disparity with peripheral fusion stimuli [53], for the associated phoria which is the amount of prism required to compensate a subjective fixation disparity [41] and for the subjective heterophoria, the vergence state without fusion [54]. These findings correspond to the general understanding that vergence shifts from eso states in far vision to exo states in near vision. This exo proximity effect refers to the group mean value that is superimposed by inter-individual variability.

However, this exo proximity effect was not found for objective fixation disparity. At least on the level of the group average, the near objective fixation disparity was practically zero with a large standard deviation and no mean change in objective fixation disparity across the viewing distances of 24, 30, and 40 cm was found. These results can be complemented by those of two other studies of our research group that used one fixed viewing distance, i. e., 60 cm in Jaschinski et al. [21] and 5 m in Schroth et al. [4]; these both studies applied similar experimental conditions (sharp central fusion stimulus, concurrent nonius adjustments, no reading saccades) and recording procedures (purpose made operation of EyeLink II, monocular calibration) so that a comparison in Fig 3B seems justified. These two mean values (and their standard deviations) at 5 m and at 60 cm resemble those of the present study between 40 and 24 cm. These three studies suggest that objective fixation disparity seems to be rather constant from far to near vision, at least for the group mean. This finding is surprising and requires a discussion in terms of methodology, previous research and physiological meaning.

First of all, the missing exo proximity effect in objective fixation disparity raises the question whether the recording methods were accurate enough for finding small changes in objective fixation disparity. This methodological issue is discussed in detail in the S2 File which provides evidence that the recording procedures would have detected an effect of viewing distance if it had existed.

The average constant level of objective fixation disparity across the viewing distances of 40 to 24 cm appears to be plausible in relation to previous research. Erkelens et al. [45] measured fixation disparity in a range of viewing distance of 91.4 to 10 cm (4 to 37 deg) with the precise search coil method in three observers who viewed light emitting diodes (LEDs) in a dim room; subjective fixation disparity was not measured. At 91.4 cm, the fixation disparity of the three subjects was—1,—5, and—7 min arc (mean = - 4.2 min arc), thus exo on the average. In the vergence range of 4 to 17 deg (i. e., 91–21 cm, including the present range of viewing distances), the slope in objective fixation disparity was—0.8 min arc / (1/meter); this would mean a negligible change of 1.3 min arc from 40 to 24 cm, i. e., the range in the present study. In Erkelens et al. [45], only at shorter viewing distances (21 to 10 cm) the slope increased to—5 min arc / (1/meter). This means that in agreement with the present results, the objective fixation disparity did not change much when targets are more distant than 21 cm; the expected exo shift in near vision became much more pronounced when targets are closer than 21 cm. However, Erkelens et al [45] generally found exo fixation disparity in their three observers, while the present study with 20 observers includes eso and exo cases across the range from 40 to 24 cm. This difference could have been the result of the selection of these particular 3 subjects in Erkelens et al. [45]. But probably the different experimental conditions were important: the sharp text characters in the present experiment are a strong stimulus for accommodation and vergence, while a light emitting diode represents a blurred star-like light source which does not well stimulate accommodation [55]. Thus, with LEDs, accommodation and vergence may tend towards their resting position which is near 1 m.

Fogt and Jones [17] investigated subjective and objective fixation disparity not as a function of viewing distance, rather the vergence state was changed by varying the absolute disparity between the images of the two eyes at a fixed 66 cm viewing distance of the display (i. e., 6 deg initial vergence). The search coil method was applied in five subjects who viewed a peripheral fusion stimulus. The increase in forced convergence had different effects in different subjects. Subject E of Fogt and Jones [17] showed the expected exo shift in both types of fixation disparity; subject C had an exo shift in objective fixation disparity and no shift in subjective fixation disparity. Interestingly, in two further subjects (B and D), both types of fixation disparity showed a slight shift in the eso direction up to a disparity offset of 5 deg reaching significantly eso states of fixation disparity at vergence angles of 11 deg; at larger convergence, fixation disparity shifted into the exo direction, as expected.

The studies of Erkelens et al. [45] and of Fogt and Jones [17] have the advantage that the objective fixation disparity curves were measured with stepwise increasing convergence over a large range of vergence. Both studies agree in the conclusion that the exo shift with increasing convergence is moderate, if any, in the range up to vergence angles of 15 deg (up to about 24 cm viewing distance); this resembles the finding in the present experimental conditions. Only at larger convergence, the objective fixation disparity shifts to strong exo objective fixation disparity. This interpretation can only be tentative since the experimental conditions varied considerably between these studies. The other two studies had a weak fusion stimulus (blurred LEDs or peripheral frame) that can be considered as weak for fusion and accommodation. The present study seems to be the first to investigate the proximity effect in subjective and objective fixation disparity for sharp central fusion stimuli. However, only a rather limited range of viewing distances of 40, 30, and 24 cm was used.

There are several other studies on objective fixation disparity at a single fixed viewing distance [29–39], but the experimental conditions differ in terms of eye tracker technology (video, DPI), calibration (monocular, binocular), experimental task (reading, dot scanning), viewing conditions (bright vs. dark background or characters); most of these studies investigated readings tasks where the fixation disparity may be influenced by the cognitive task or the position of fixation across the line of text. Since the experimental conditions differed in many respect, it was not possible to explain, why these studies differed considerably in the level of group mean objective fixation disparity, i.e., eso, zero and exo.

To summarize, the general and typically expected exo vergence state in near vision is reflected in heterophoria and subjective fixation disparity, but not in objective fixation disparity, at least for the group mean values. The most likely explanation is that the accommodative effort in near vision shifts the objective fixation disparity into the eso direction in some subjects. This is supported by two observations: (1) the study of Erkelens et al. [45] found exo fixation disparity with weak accommodative stimuli while the present study used sharp stimuli, thus stronger accommodative stimuli and found an average objective fixation disparity of zero, (2) in the present study, observers with higher accommodative performance tended to have a more eso objective fixation disparity, which may shift the objective fixation disparity distribution into the eso direction. In contrast, the values of subjective fixation disparity were nearly all exo and were not correlated with the inter-individual variance in accommodative performance, neither in the present study nor in Jaschinski [43]. This suggests that the inter-individual distribution of objective fixation disparity (but not of subjective fixation disparity) may be affected by the observer’s accommodative performance. Note, that within an individual, the modulation of accommodation (e. g. by applying spherical lenses) will affect fixation disparity [25].

### Subjective versus objective fixation disparity

This discussion describes the—very different—physiological mechanisms of the two types of fixation disparity and proposes a model for the relation between these two.

The objective fixation disparity is the vergence error relative to the reference condition that was determined during the calibration, i.e., the monocular fixation of the calibration targets in the left and right eye. Monocular fixation points are assumed to be projected onto the centre of the foveola. But note (1) that this assumption cannot be proven by eye tracking procedures and (2) this zero point of eye position refers to the average across fixation eye movements that always occur. An objective fixation disparity means that the binocular fixation targets have a retinal disparity due to an oculomotor vergence error.

Despite an objective fixation disparity of up to some tens of minutes of arc, double vision does not occur. This is achieved by sensory fusion, also referred to as Panum’s fusion mechanism: identical parts in the two retinal images (binocular stimuli) receive the same visual directions (single vision), although they are projected on non-corresponding retinal points. Fusion will not occur, if two monocular stimuli differ between the two eyes with respect, e. g., to size or spatial structure. In the present dichoptic nonius test, fusion is achieved for the two identical binocular stimuli (i. e., the central text characters XOX), but how are the non-fusible dichoptic nonius lines processed? Theoretically, one may consider two extreme conditions. In a first condition, one could expect that dichoptic nonius lines were processed in the same way as the adjacent binocular stimuli since fusion is local in a certain retinal area where also monocular targets receive the visual direction of nearby binocular targets (see Introduction); then, nonius lines should be perceived in line. This can occur in some observers and some test conditions, but in many cases physically aligned nonius lines are perceived with an offset, so that–in general–the first condition does not apply. In a second condition, one may expect that the fusion system affects only identical stimuli in the two eyes and not the non-fusible nonius lines; then, the nonius lines should indicate exactly the objective fixation disparity. This second condition generally also does not apply, since the subjective fixation disparity is typically much smaller than objective fixation disparity. It seems as if an intermediate condition applies since subjective fixation disparity ranges between zero and objective fixation disparity in most cases. The fusion mechanism shifts the visual directions of the dichoptic nonius lines by a smaller amount than the binocular targets. For this mechanism, stimulus conditions play a role [18]: the further away the nonius lines are from the fusion stimulus, the more the amount of subjective fixation disparity increases and approaches the objective fixation disparity (however, peripheral nonius judgements are less reliable).

These observations lead to the following hypothetical mechanism: retinal disparity stimulates an oculomotor vergence movement so that a motor vergence error (objective fixation disparity) may remain. This objective fixation disparity is the stimulus for the sensory fusion mechanism of the binocular target (shift in visual direction by the full amount of objective fixation disparity). Objective fixation disparity may also be the stimulus for the shift in visual direction of the dichoptic nonius lines; the latter shift is–in most cases—smaller than the shift of the visual directions of the binocular targets. More formally and in the most simple way, subjective fixation disparity of an observer i would be a percentage of objective fixation disparity, i. e., sFD_i_ = c_i_ * oFD_i_, with a factor c_i_ that ranges between 0 and 1. This simple model has the following features: (1) the ratio sFD_i_/oFD_i_ depends on the individual which is suggested since each type of fixation disparity can be measured reliably with a test-retest correlations around r = 0.7, but the inter-individual correlation between the two types of fixation disparity is typically only r = 0.5, (2) A zero sFD is associated with a zero oFD, (3) all properties of objective fixation subjective would apply to subjective fixation disparity since the motor objective fixation disparity is the stimulus for the sensory mechanism. This reflects that both types of fixation disparity are correlated with heterophoria [21]; both shift in the exo direction with blurred stimuli [56, 57] and both are–more or less–similarly affected by changing the absolute disparity [17].

Testing the hypothesis sFD_i_ = c_i_ * oFD_i_ requires repeated measurements of sFD and oFD per individual. These are available in the literature in the two studies of Kertesz and Lee [11] and Fogt and Jones [17] although these authors did not propose or test this model explicitly. These studies applied forced vergence which leads to a large intra-individual variation in fixation disparity. A re-analysis of their published data of 9 subjects of the two studies showed that in 8 subjects the two types of fixation disparity are well correlated (median r = 0.95, range 0.77 to 0.99); the sFD/oFD ratio had a median of 0.58 (range 0.26 to 0.71); one subject in Fogt and Jones (1998) had a smaller correlation (r = 0.3), probably due to a small variance in sFD which prevented to show up correlations. Thus, within most individuals, sFD appears as a rather fixed percentage of oFD, at least in the condition when fixation disparity is varied due to forced vergence, i. e., with a discrepancy between vergence and accommodation.

This model sFD_i_ = c_i_ * oFD_i_ was also tested with the data of the present study, where vergence is changed by varying the viewing distance with corresponding vergence and accommodation. In this condition the fixation disparity varies much less as if vergence is changed by varying disparity; this was shown for subjective fixation disparity as a function of prism load or viewing distance [42]. The small variability due to viewing distance, both in sFD and oFD might be the reason why the individual correlations between oFD and sFD were small and insignificant in many cases, some single subjects had positive relations, others also large negative correlations. Thus, in the present study sFD cannot be explained by oFD on the individual level.

Further, the model sFD_i_ = c_i_ * oFD_i_ has the consequence that one should expect an inter-individual correlation with a regression line having a zero intercept and slope smaller than 1. Such an inter-individual regression was tested in two earlier studies. Jaschinski et al. [21] found such a correlation of about 0.5 in sample with predominantly exo objective fixation disparity at a 60 cm viewing distance. Schroth et al. [4] applied far vision and reported that the correlation tended to be higher in observers with an eso associated phoria in far vision (i. e., a base-out aligning prism). In the present near vision study, the subgroup with exo oFD showed the hypothesized inter-individual regression (Fig 6A), but not the subgroup with eso oFD (Fig 6B). Thus, it seems as if an eso oFD in near vision is a condition where the opposite exo sign of sFD occurs in 7 of these 8 cases; thus the simple model of sFD_i_ = c_i_ * oFD_i_ does not apply, at least not with positive values of c_i_. Another discrepancy occurred in the group mean values: if, according to the above model, sFD would be a group mean percentage of oFD, the sFD proximity curve would follow the oFD curve. But, this seems not to be the case, due to the significant slope in sFD and the constant level in oFD (Fig 3). However, the interpretation of Fig 3B may only be preliminary since the experimental evidence for the objective proximity curve is sparse: it was not yet measured over the complete range of viewing distances in the same sample of subjects.

Thus, it appears that the simple model sFD_i_ = c_i_ * oFD_i_ can only be a working hypothesis that was confirmed in some conditions, but deviations occurred in other conditions. The reason for these discrepancies could not yet be clarified with the data presently available. It seems as if a general simple model or mechanism does not exist. Rather the relation between subjective and objective fixation disparity may depend on the viewing distance (far versus near vision) and individual dispositions of fixation disparity (eso versus exo). The dichotomy between eso and exo fixation disparity may not simply be a different direction in geometry or sign in mathematic terms, but reflect different physiological mechanisms [4].

### Benefits, limitations and perspectives of the study

This study is the first to investigate objective fixation disparity in a–relatively–large sample of 20 observers who viewed a display in near vision with adequate stimulation of vergence and accommodation. Frequent repeated measurements and replication of results in two sessions allowed reducing random measurement error in recordings with a video eye tracker. In this way, individual differences in objective fixation disparity could statistically be confirmed with mixed-effects models. This is an extension of previous research that was confined to only few participants in the earlier studies on objective fixation disparity (mostly using invasive scleral search coils. The approach of the present study has the potential (1) to add to vergence research the systematical focus on individual differences in order to clarify the underlying physiological mechanisms and (2) to include objective recordings of fixation disparity into the diagnostic procedures of clinical optometric, at least if a more elaborate investigation might be indicated.

The limitations include the small range of viewing distances that were applied. Also the potential role of accommodation could not fully be accounted for. This may be overcome in future studies which may also try to elucidate the sensory processing of dichoptic nonius lines; therefore, the relation between subjective and objective fixation disparity should be tested within individuals in different conditions of vergence load, i. e., both in forced vergence and in natural vergence.

## Conclusion

For heterophoria and subjective fixation disparity, this study replicated that the binocular system does not properly adjust to near targets: outward (exo) deviations typically increase as the viewing distance is shortened. This exo proximity effect—however—was not found for objective fixation disparity, which–on the average–was zero. But individuals can have reliable outward (exo) or inward (eso) vergence errors. Cases with eso objective fixation disparity tend to have less exo states of subjective fixation disparity and heterophoria.

In summary, the two types of fixation disparity seem to respond in a different way when the viewing distance is shortened. Motor and sensory fusion–as reflected by objective and subjective fixation disparity–exhibit complex interactions that may differ between individuals (eso versus exo) and vary with viewing distance (far versus near vision).

## Supporting Information

S1 Fig Complete dataset of the individual subjects. For each participant it is displayed the robust regression line of subjective and objective fixation disparity as a function of the vergence stimulus (1/meter). For each vergence stimulus, each data point refers to one of the 8 single runs of both sessions (see Fig 4).
(TIF)

S1 File Data analyses and mixed-effects models. (PDF)

S2 File Accuracy of objective fixation disparity. (PDF)
