# Supplementary figures and images for: Individual Objective and Subjective Fixation Disparity in Near Vision
Source: PLoS One. 2017 Jan 30;12(1):e0170190. doi: 10.1371/journal.pone.0170190 (PMC5279731; doi:10.1371/journal.pone.0170190)

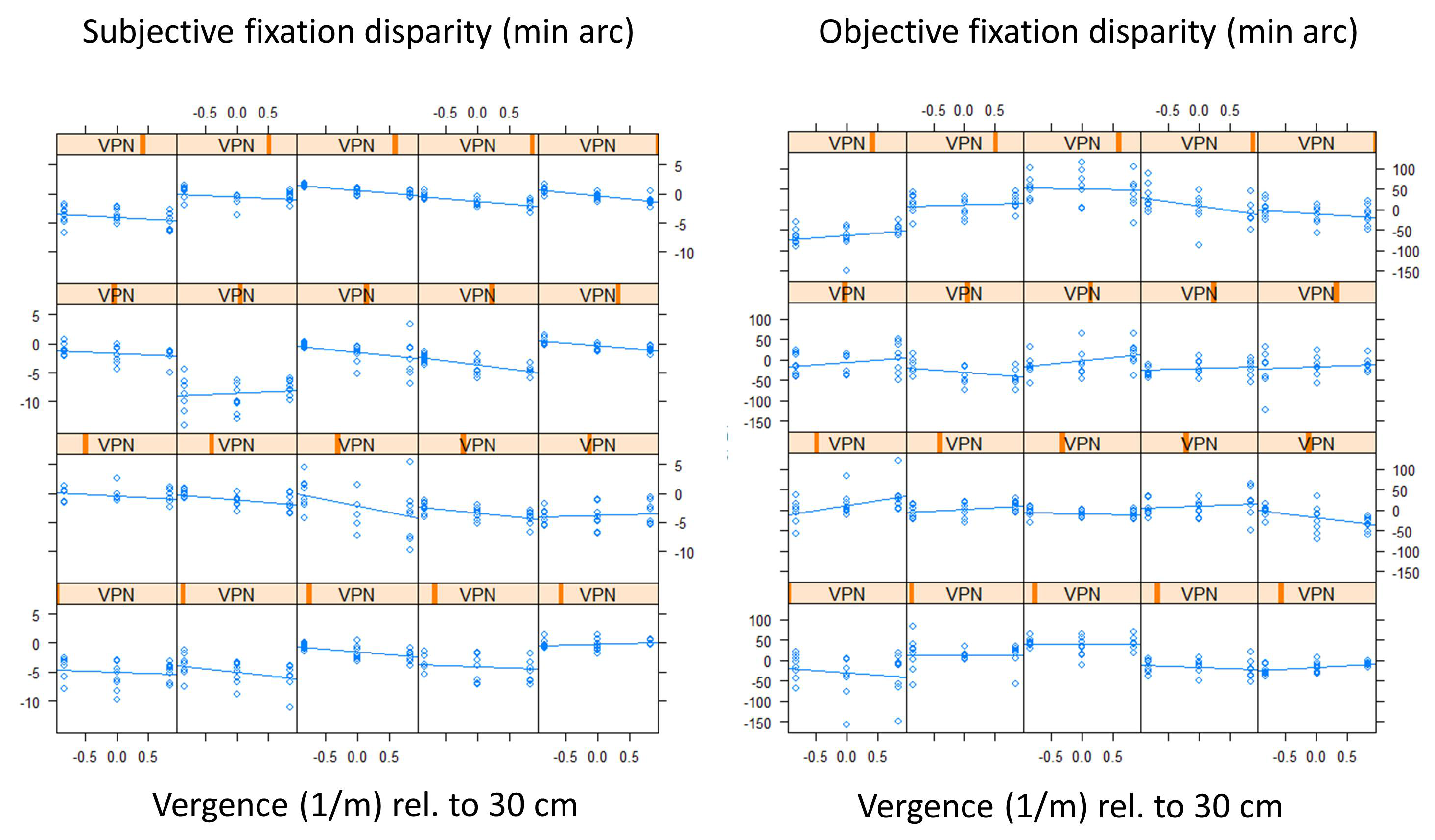

Supplement: S1 Fig — For each participant it is displayed the robust regression line of subjective and objective fixation disparity as a function of the vergence stimulus (1/meter). For each vergence stimulus, each data point refers to one of the 8 single runs of both sessions (see Fig 4). (TIF) [file pone.0170190.s001.tif]
