# Supplementary material for: Individual Objective and Subjective Fixation Disparity in Near Vision
Source: PLoS One. 2017 Jan 30;12(1):e0170190. doi: 10.1371/journal.pone.0170190 (PMC5279731; doi:10.1371/journal.pone.0170190)
Supplement: S1 File — (PDF) [file pone.0170190.s002.pdf]

Supporting information

S1 File. Data analyses and mixed-effects models

Within the one minute recording period of a single run, the observers typically made n = 10 - 20 single nonius adjustments. This number depended on how fast the observer adjusted the nonius lines. Thus n single data per run were available of both subjective and objective fixation disparity and of pupil size. These single data per run were used for a regression of objective fixation disparity versus pupil size, in order to find an estimation of objective fixation disparity that is free from the pupil artifact in eye movement recordings using video eye trackers. This regression procedure implies an averaging across variability within the one minute run. The details of these procedures are described in Jaschinski (2016) [46] for the present data set. For subjective fixation disparity, the n single nonius adjustments per run were averaged. All following analyses are based on one data point per run.

S1 Fig shows the scatter plot of subjective and objective fixation disparity as a function of viewing distance (Stim), separately for each individual. At each viewing distance (40, 30, 24 cm) all available eight data points of the two sessions are included (from the four runs per session). These plots show more outliers for objective than for subjective fixation disparity. Most likely, these outliers are measurement errors since objective fixation disparity is at the limit of the accuracy of video eye trackers.

The influence of measurement error was reduced by averaging the data of one session in order to arrive at a better estimation of the individual fixation disparity per session. This procedure is adequate for the present study, which investigates the inter-individual differences by correlating the intra-individual measures of fixation disparity within the sample of 20 observers. The idiosyncrasy of fixation disparity was tested by a random effect in mixed-effects models, by Bland-Altman-analyses and by test-retest correlations between the two sessions. Further, all results were tested in both of the two sessions.

In mixed-effects models, individual differences are analyzed by a random factor of subjects: the model estimates an inter-individual standard deviation SD_{inter} between subjects

(the intercept subject effect) and an intra-individual standard deviation SD_{intra} within subjects. The latter SD_{intra} was estimated by the Session-subject effect which described how the data vary within a subject from session to session. In the statistical software R, SD_{intra} and SD_{intra} result from combined random effects of Intercept and Session: (1 + Session|Subject). We have applied this procedure in two earlier studies [21, 52]. The ratio $R_i = SD_{inter} / SD_{intra}$, the repeatability index, is a measure of how strongly a measure varies between subjects, in relation to how strong it varies within subjects, e. g. between sessions. This procedure averages across the variability within a session – either due to measurement error or due to physiological variability.

As described in the main manuscript, the most appropriate mixed-effects models differed in structure for the three dependent variables heterophoria, subjective and objective fixation disparity. These most appropriate models are formulated in LMER as follows:

```
het ~ Distance + (Distance | Subject) + (1 + Session | Subject)
sFD ~ Distance + (1 + Session| Subject)
oFD ~ (1 + Session | Subject)
```

Results of random subject effects also depend on the sample size: in small sample sizes, it will be unlikely to have subjects with extreme values of fixation disparity, thus SD_{inter} will tend to be smaller than in large samples; the present sample of 20 observers is much larger than most earlier studies in this field (see [21]) and resembles the sample size of n = 24 in [4].
