# Supplementary material for: Individual Objective and Subjective Fixation Disparity in Near Vision
Source: PLoS One. 2017 Jan 30;12(1):e0170190. doi: 10.1371/journal.pone.0170190 (PMC5279731; doi:10.1371/journal.pone.0170190)
Supplement: S2 File — (PDF) [file pone.0170190.s003.pdf]

Supporting information

S2 File. Accuracy of objective fixation disparity

Measurements of fixation disparity below about 1 deg are a challenge for video eye trackers. The physical noise limited resolution of the EyeLink II system is below 1 minutes of arc (as specified by the manufacturer), while the empirical resolution in real eye movement recordings is larger due to physiological variability, head instability, or calibration errors. The recording procedures had been refined in our laboratory over many years in order to reduce measurement errors. The quality of the recordings can be described by the following findings.

In a previous study [58] we had evaluated the empirical resolution of our recording procedures: changes in eye position of about 4 – 6 min arc could be resolved, as average of 29 to 41 single eye position recordings. Our recent study [46] of the pupillar artefact on fixation disparity included Bland-Altman analyses showing that the standard deviation of the difference between fixation disparity of the two sessions was about 19 min arc at the 40 cm viewing distance (this standard deviation increased - for physiological reasons - as the viewing distance was shortened). The test-retest correlations between the two sessions were 0.78, and 0.71, and 0.51 for the fixation disparity corrected for the pupillary artefact, at the viewing distances of 40, 30, and 24 cm, respectively.

Thus, the present eye tracker procedures are adequate for this research which aims to identify – despite intra-individual variability - the mean fixation disparity of an observer as an individual characteristic and to investigate its physiological properties.

In the present study, the missing effect of viewing distance on objective fixation disparity raises the question whether the recording methods were accurate enough for finding small changes in objective fixation disparity. The following arguments can be made.

(a) individual differences in objective fixation disparity could be identified by a significant random subject factor in the mixed-effects model and by a high test-retest correlation between sessions,

(b) these individual values of objective fixation disparity showed a physiologically expected correlation with two other vergence measures (subjective fixation disparity and heterophoria) where no methodological caveats exist,

(c) Our laboratory had identified changes in objective fixation disparity by a few minutes of arc due to blurring the stimulus or by modifying the spatial structure of text characters as shown in [56, 59].

These arguments suggest that changes in objective fixation disparity would have been detected if they would have existed.

A particular result was the observation that 8 observers had a reliable eso oFD while the sFD was exo in 7 of these observers. These different directions of the observed fixation disparity are surprising given the general – although only medium – correlation between sFD and oFD. One may ask whether the oFD values were generally shifted into the eso direction by about 50 min arc due to some measurement artifact of the present procedure, which Fig 5 and Fig 7B may suggest. This is unlikely since the cases with exo oFD (Fig 6A) showed the expected positive correlation with sFD and an intercept close to the intercept as expected and in agreement with the proposed model $sFD_i = c_i * oFD_i$ (see below). If all oFD values were shifted towards eso by 50 min arc due to an artifact that may apply generally to all observers, then the intercept in the exo group in Fig 6A would strongly deviate from the origin. Moreover, the observers with eso oFD values are not random in the sample: rather, they have small negative values for both heterophoria and sFD and tend to have a larger accommodative performance.

A quantitative description of the accuracy of objective measures of fixation disparity can be given be the individual standard errors of the mean (SEM) as they result from the robust regression of fixation disparity as a function of vergence (see Fig 3). This robust regression was made for each subject and each session separately and provides a measure

of the y-intercept (i. e., the estimated fixation disparity at 30 cm viewing distance) and the slope (i. e., change in fixation disparity with viewing distance). For these both coefficients, the robust regression lmrob of the statistical software R provides a standard error of the mean that is given in Table S2 for the three dependent variables heterophoria, subjective and objective fixation disparity. These standard errors of individual mean values apply to the data points in the correlation scatter plots in Fig 5, 6, 7 and 8 where the SEM can be expressed as a certain percentage of the dot radius. For heterophoria, this percentage was about 81 %, thus the average SEM is slightly smaller than the dot radius. For subjective fixation disparity this percentage was about 100%, so that the dot size resembles a range of ±1 SEM, and for objective fixation disparity the percentage was about 170%, thus the range of ±1 SEM is slightly less than twice the dote size. These percentages refer to the average participant; the group standard deviations of the SEM is given in Table S2. It can be concluded that these standard errors are much smaller than the inter-individual range of the dependent variable, thus individual differences can be determined.

References

58. Jainta S, Hoormann J, Jaschinski W. Accommodation modulates the individual difference between objective and subjective measures of the final convergence step response. Ophthalmic & Physiological Optics. 2009;29(2):162-72. DOI: 10.1111/j.1475-1313.2008.00624.x
59. Jainta S, Jaschinski W, Wilkins AJ. Fixation disparity during reading is affected by periodic letter strokes within a word. Perception. 2010;39:36-. PubMed PMID: WOS:000289740100107.

Table S2. Standard error of the mean (SEM) of individual coefficients of the robust regression. As shown in Fig 4, the robust regression provides the y-intercept and slope and also the corresponding standard errors for each individual and session. These SEM-values are described here by their group means and group standard deviations for each dependent variable, i. e. heterophoria, subjective and objective fixation disparity it is displayed.

		Estimated value at 30 cm viewing distance	Slope as a function of viewing distance
		SEM<br>Group mean ± SD	SEM<br>Group mean ± SD
Heterophoria (deg)	Session 1	0.18 ± 0.06	0.27 ± 0.09
	Session 2	0.16 ± 0.07	0.23 ± 0.10
Subjective fixation disparity (min arc)	Session 1	0.42 ± 0.21	0.62 ± 0.32
	Session 2	0.36 ± 0.19	0.53 ± 0.28
Objective fixation disparity (min arc)	Session 1	6.76 ± 2.77	9.92 ± 4.09
	Session 2	6.81 ± 3.11	9.96 ± 4.52
